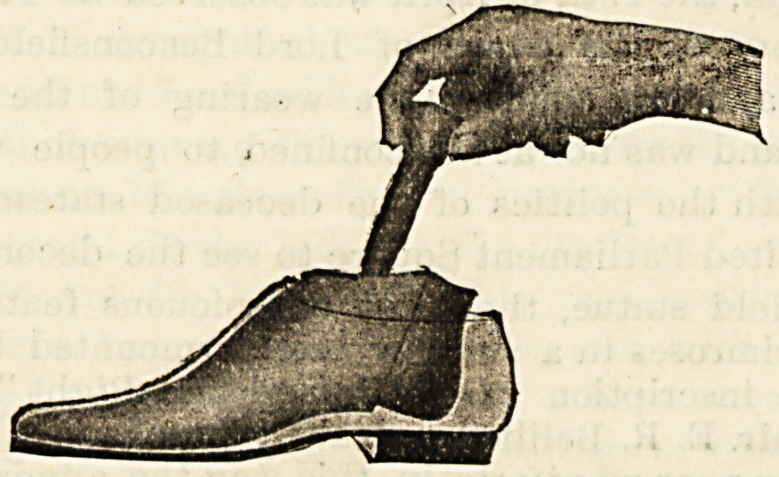# The Hospital. Nursing Section

**Published:** 1902-04-26

**Authors:** 


### The Hospital.

### Nursing Section.

Contributions for this Section of "The Hospital" should be addressed to the Editor, "The Hospital" Nursing Section, 28 & 29 Southampton Street, Strand, London, W.C.

No. 813.—Vol. XXXII. *SATURDAY, APRIL 26, 1902.*

### Notes on News from the Nursing World.

### QUEEN VICTORIA'S JUBILEE INSTITUTE FOR NURSES.

In submitting the report for the year 1901 of Queen Victoria's Jubilee Institute for Nurses to her Majesty, Queen Alexandra, the council refer to the reception of the Queen's nurses at Marlborough House last July. They express their confidence that "this Royal recognition of those who are so unremittingly giving the best of their abilities and knowledge to aid the sick poor must tend to raise the work of the institution in public estimation," and that "the gracious words of sympathy and appreciation spoken by her Majesty to the nurses will deepen their devotion to their work." Attention is called to the statistics at the end of the report, from which it appears that the total number of affiliated associations employing Queen's nurses on December 31st, 1901, was 521, as against 484 on December 31st, 1900; a net increase, after allowing for withdrawals, of 37, of which 16 were in England, 13 in Scotland, seven in Ireland, and one in Wales; that the total number of Queen's nurses on the Roll was 858, as against 794, an increase of 64, of whom 30 had rejoined the institute after having withdrawn for a longer or shorter time; and that the number of nurses who had resigned during the year, out of a larger number employed, had been one less than in the previous year. Alluding to the actual work done by the Queen's nurses, the report says, "the witness that is constantly borne to their kindness and skill by the poor is very frequent and most gratifying." In the accompanying supplementary report by the council and the committee of the Queen's Commemoration Fund, allusion is made to the difficulty in finding suitable nurses for training as Queen's nurses, which it is stated "has in no way diminished," and the council again urge upon the committees of affiliated associations the desirability of raising, wherever possible, the nurses' salaries, so that the services of those already trained may be retained.

### PRINCESS BEATRICE AT RYDE HOSPITAL.

Princess Henry of Battenberg, President of the Royal Isle of Wight County Hospital at Ryde, paid a visit to the hospital during the afternoon of Friday, the 18th inst. The Princess was received at the entrance door by Rev. W. H. E. Welby, chairman of the committee, Mr. Buck, the senior member of the medical Staff, Miss Antram, the matron, and Mr. R. C. H. Kennedy, the house surgeon. The sisters in turn were duly presented to Her Royal Highness as she visited the wards of the main building, and the Queen Victoria ward for Children. The Princess asked many questions as to the progress of the patients and addressed several of them individually. Before leaving she most kindly gave some pictures to be placed in the children's ward, where one big boy of eight months pleased her greatly by his winsome loving manner. Charming flowers were prettily arranged in the wards and corridors, and Her Royal Highness expressed herself much interested with all she saw. A visit was also paid to the Milligan Convalescent Home, where Miss Yate, the matron, was presented, and after signing the visitors' books, the Princess took her departure amid the respectful salutations of patients who were enjoying the fine air in the garden which surrounds the hospital. The Royal visit lasted about an hour and a quarter.

### AMBULANCE DUTY ON CORONATION DAY.

Several inquiries have reached us from trained nurses who wish to know "whether extra nurses will be needed for ambulance duty during the Coronation festivities." It may be hoped that the ambulance work on the occasion will not be serious, but it will be very necessary to be prepared for all emergencies, and we have no doubt that the Secretary of the St. John Ambulance Association, St. John's Gate, Clerkenwell, E.C., will be glad to hear from any nurses who are willing to render assistance on the occasion.

### THE WAR NURSES.

The *City of Vienna* arrived from South Africa on the 15th inst., and the following nursing sisters disembarked at Southampton:—E. Johnson, M. Dowker, A. Jones, and F. E. Thomas, all requiring one month's leave before returning to South Africa, and F. S. Holcroft, invalided home, but wishing to return after sick leave. All are members of the A.N.S.R. The *Orcana* arrived April 16th. There were on board: E. M. Hayden, K. F. Grosvenor, and F. H. Barry, all of whom will rejoin the ship, and F. W. Carner, invalided home, but returning to South Africa after three months' leave, which has been granted. All are members of the A.N.S.R.

### THE MALE NURSE IN SOUTH AFRICA.

The conclusion drawn by Mr. Francis Fremantle as the result of his experiences in South Africa during the time he acted as civil surgeon to His Majesty's forces, is that the nursing at general and stationary hospitals in time of war should be almost entirely carried out by nursing sisters; while in order to provide for contingencies at home he would like to see a small staff of male nurses trained and employed at the large civil hospitals in the accident wards, for use as a reserve for the rougher nursing work. Mr. Fremantle's experience of male nurses is so unsatisfactory that he says "the nursing of bedded hospitals even at advanced bases should be done by women nurses."

### NURSES AMONG CANNIBALS.

The Bishop of New Guinea, who has arrived in England for the purpose of soliciting funds for his mission in the island, declares that one of the most important agencies is the nursing branch. There are four trained nurses at work in the diocese, and the testimony of Dr. Stone Wigg is that they are most devoted. They run some risk, too, of an unusual danger, which is enough to strike terror into the boldest of hearts. Cannibals have not yet been exterminated in New Guinea, and during last year not only the veteran missionary, James Chalmers, but three other white men also were killed and eaten, as well as several natives. The Bishop has in his possession the jawbone of a native boy who was eaten so recently as September. It is naturally among the cannibals that the missionaries most desire to make converts, but their existence has to be taken into account by those who undertake nursing.

### DEATH OF A NIGHTINGALE NURSE IN AUSTRALIA.

We regret to learn of the death of Miss Elizabeth Graham Sherlock, one of the best-known nurses in Australia. Miss Sherlock was trained at the Nightingale Home, and was subsequently, in the mother country, staff nurse at the Royal Infirmary, Edinburgh, superintendent-nurse at Fusehill Hospital, Carlisle, and matron of Carlisle Fever Hospital. In 1886 she went to the Antipodes, and did private nursing in Sydney for a time, afterwards becoming successively matron at the Armidale and New England Hospital, Armidale, matron of the Children's Hospital and Benevolent Asylum at Rockhampton, matron of Junee Hospital, matron of the Hospital and Benevolent Asylum, Newcastle, matron of the Hill End and Tambaroora Hospital, matron of the Carcoar District Hospital, and finally matron of the District Hospital at Goodooga. She was greatly esteemed at all the different and important institutions with which she was in turn associated, and her decease, after a short but painful illness, is much lamented. One of her last requests to the honorary secretary of the Goodooga Hospital, when she realised that the end was near, was to ask him, as a favour, to forward a notice of her death and her testimonials to The Hospital.

### WORKHOUSE MASTERS IN CONCLAVE.

There has been a conference of members of the District Association of Workhouse Masters and Matrons at Ipswich to consider "the difficulty, and its causes, in obtaining qualified nurses in provincial and country workhouse infirmaries, and the responsibilities and duties of the superintendent nurse." Obviously, the workhouse masters and matrons have a perfect right to do their best to protect their own interests, and with the tone of the speeches at Ipswich there is no fault to be found. We are glad to see that several of the speakers very warmly advocated suitable recreations and reasonable comforts for the nurses in their leisure time, and that no hard words were used. But on the question of principle raised by the Chairman, Mr. Grayson, of Ipswich, who made the most important speech, and obtained the support of many who attended the conference, it must be said that he adopted an attitude of aggressive hostility to the reforms which Miss Gibson recently advocated with such conspicuous ability. Mr. Grayson, who has evidently been alarmed by the strong case presented by the matron of Birmingham Infirmary in favour of investing the superintendent nurse with supremacy, has apprehensions of the day when the untrained matron, with authority over trained nurses, will be no more. We hope that it will come sooner than Mr. Grayson fears, and the fact that he endorses the reactionary demand of the Atcham Union, which, we learn, has been urged before the Departmental Committee on Nursing in Workhouses by the Master of Shrewsbury Workhouse, will scarcely delay it. It is not surprising that from his point of view Mr. Grayson insists that the master of a workhouse should have absolute control over the whole establishment, including the infirmary, and that the matron, whatever her training or absence of it, should have the supervision of "all female officers and their work," including, of course, the superintendent nurse. In the adoption of this policy lies, no doubt, the only hope of those who are opposed to the reform of nursing in workhouse infirmaries. But we do not believe for a moment that it will be allowed to prevail.

### THE NEW INFIRMARY AT SHIRLEY WARREN.

A feature of the handsome new infirmary at Shirley Warren, which has just been opened under the auspices of the Southampton Board of Guardians, is a separate nurses' home with a tennis lawn in front. The building itself, which stands on the top of a rather steep hill, in prettily laid-out grounds, has three male and two female blocks, which, according to a correspondent who attended the opening ceremony, are to be admirably equipped. One of these is already furnished as the others are to be, and the marble-topped lockers, stoves, and other articles, our correspondent states, give the place a uniform and thoroughly good appearance. Other portions of the building are a maternity block, an isolation block, a laundry, and a mortuary, as well as a compact administrative block. The interior decoration is pleasing, pale sage green walls with a warm red dado. The covered ways, our correspondent says, are like cloisters, lead to all five blocks, and connect them with the administrative centre, which includes a very fine kitchen. The matron and sisters of the new infirmary are, we understand, London-trained nurses. Altogether, the Southampton Guardians deserve hearty congratulation upon the result of their efforts to meet the requirements of the times.

### NURSING AT CAMBRIDGE INFECTIOUS DISEASES HOSPITAL.

The Cambridge Town Council had under consideration at their meeting last week a number of serious charges reflecting on the management of the Infectious Diseases Hospital, locally known as the Sanatorium. These charges were dealt with at great length by Dr. Dalton, the Chairman of the Public Health Committee, who at the close of his speech moved "That this council has every confidence in the matron and nurses at the Infectious Diseases Hospital." But in the course of his speech he made the following admissions:—That on more than one occasion two nurses had omitted to wash the thermometer after it had been used by a patient, and had wiped it with a towel; that a nurse had omitted to give her patient medicine once during the most acute part of the illness; and that the temperature of the large scarlet fever ward of the female patients was on one occasion in the early morning below 60° owing to insufficient fires, or the coal not being up to sample. With regard to one complaint, the language of Dr. Dalton was a little vague. He observed that "as to the child who was discharged with nits in her head, it was more than three years since a complaint of that kind had been made, though there had been complaints under previous matrons." Clearly this charge was sustained. In view of the gravity of some of the other allegations which were preferred against the management, the conclusions of the Public Health Committee, and their endorsement by the Town Council—26 of whom voted for Dr. Dalton's motion and three against—must be regarded as satisfactory by the matron and nurses. To this it may be added that while a medical practitioner in the town who had patients in the scarlet fever block complained of several irregularities, Mr. Digby, a member of the Corporation who had had five children in the hospital, and lost one, declared that having been in the wards morning, noon, and night, he had "the utmost confidence in the institution." But the admissions of the chairman of the Public Health Committee show, at any rate, that there is room for considerable improvement in the nursing arrangements, and that the extra charge nurse, whose appointment the committee recommend, is badly needed. As to the future, we hope that the matron will fully justify the faith which the Town Council repose in her judgment and capacity.

### FUNERAL OF MISS SHIRLEY.

The mortal remains of Miss Elizabeth Mary Shirley, lady superintendent of the Staffordshire Institution for Nurses, whose death we announced last week, were laid to rest on the 15th inst. at Hartshill Cemetery. A large crowd, consisting largely of the working class, by whom she was greatly esteemed, assembled, and in spite of a continued downfall of rain, lined the route to Hartshill Church, where the first part of the service took place. The coffin was covered with handsome wreaths, and most of the mourners, as well as 70 nurses, carried with them some floral emblem of respect to place on the grave. The Bishop of Shrewsbury read the burial service, and several of the clergy were present, the ceremony throughout being of a most impressive character. Miss Shirley was a member of the Royal National Pension Fund, and a representative of the policy holders. In this capacity she acted on the Committee of Junius S. Morgan Benevolent Fund.

### CROYDON GUARDIANS THREATEN THE LOCAL GOVERNMENT BOARD.

At a meeting of the Croydon Guardians last week Mr. G. J. Allen moved: "That the Clerk be instructed to address a communication to the Local Government Board expressive of the surprise of the Guardians that no notice has been taken of their further communications of January 31st and February 5th last on matters relating to the administration of the infirmary, and requesting that the guardians may be furnished with the decision of the board on the several matters therein referred to at an early date." He said that the manner in which the Croydon Board was being treated by the Local Government Board was not creditable to the Local Government Board. "He, for one, if the Local Government Board did not support the guardians, would not condescend to hold a seat under them, and should retire. The Local Government Board should have courage to say whether they wished for an inquiry or not. The Board seemed to think that by keeping quiet they would weary the Croydon Board. It was absolutely certain that the members of the infirmary committee would never submit to bad administration in the infirmary without perhaps taking rather stronger measures than they had done in the past. The Croydon Board had asked for an interview with the Local Government Board, and this had not been granted, and they had not had the courtesy to reply." Mr. J. A. Try, in seconding, said that the time had arrived when something must be done. Mrs. Williams advised the board to fix a date for the reply to be received, after which they would retire in a body.

### DUBLIN NURSES' CLUB.

The concluding lecture for the session 1901 and 1902 was delivered at the Nurses' Club, Stephen's Green, Dublin, on Tuesday evening last week by Dr. Coleman, Physician to the Richmond Hospital, on "The Enteric Fever in South Africa." The lecture, which was most interesting, was largely attended, and was listened to by some who had themselves been on duty at the seat of war. At its conclusion a hearty vote of thanks to the lecturer was unanimously passed. It is felt that the series of lectures has been of great benefit, and it is hoped that the club will receive a large accession of members during the present year.

### SICK-ROOM COOKERY.

During the past winter session thirteen nurses from the Bradford Royal Infirmary, six nurses from the Children's Hospital, and six nurses from the Union Hospital at Bradford, have attended a course of lectures on sick-room cookery, given by Miss Maude Mason, at the Bradford School of Cookery. The lessons were so arranged that two-thirds were demonstrations, and one-third practical lessons. At the conclusion of the course it was decided that they should have certificates granted to them, and in accordance with this resolution the certificates were presented to them on Wednesday, April 16th, by Mrs. Alfred Illingworth. A vote of thanks to Mrs. Illingworth was proposed by Miss Mason and seconded by Mrs. Magill, the matron of the Royal Infirmary. Mrs. Magill remarked that although some people thought it unnecessary to teach cooking to nurses, she was fully convinced that it was essential that it should be included in a nurse's training. They did not need it so much while they were in hospital, but it must be remembered that the greater proportion of nurses eventually went to nurse in private houses or in nursing homes where they were much more valuable if they had a knowledge of cooking.

### SHORT ITEMS.

The Jubilee Bazaar in aid of the Hospital for Sick Children, Great Ormond Street, is to be held in the Royal Botanical Gardens, Regent's Park, instead of the hospital gardens, which would be too small for the proportions to which the scheme has now grown. It will take place during the first week in July, and the nurses of the hospital will have a stall where they will sell sweets, etc.—The quarterly meeting of the General Council of the Royal British Nurses' Association will be held at 10 Orchard Street on Friday, April 25th, at 5 p.m., her Royal Highness Princess Christian, the President, in the chair.

### Lectures on Gynæcology for Nurses.

By Robert Jardine, M.D., M.R.C.S., F.F.P. and S G., F.R.S.E., Senior Physician to the Glasgow Maternity Hospital, Examiner in Midwifery to the University of Glasgow.

### X.—VESICO-VAGINAL FISTULA.

In this condition an opening exists between the vagina and bladder. It is generally the result of sloughing after a severe labour, 'and in a few cases it may be caused by accidental perforation of the bladder with instruments, or by a sharp spicule of bone during the operation of craniotomy. The pressure of an ill-fitting pessary might also cause it. The most hopeless ones are, however, caused by cancer opening into the bladder by destroying the wall.

The patient will complain that her urine dribbles away from her continually. In a very short time the external genitals and thighs will become sodden, red, and excoriated, and she will suffer greatly from irritation of the parts. The parts will become very septic, and the bladder will soon become inflamed from septic cystitis. If the condition has arisen after labour the opening into the bladder will not usually be established until the bruised parts have had time to slough, so it will be well on in the second week of the puerperium before the urine begins to flow. Of course, in a case of accidental perforation of the bladder with instruments or a spicule of bone, the opening will be established at once.

*Diagnosis.*—A continuous flow of urine "always" makes one suspect that there may be a fistula, but there are two other conditions in which this occurs. The first is incontinence of urine, and the second is the very opposite, viz., retention of urine with an overflow. In incontinence the sphincter of the bladder, that is the little circular muscle at the neck of the bladder which under ordinary conditions retains the urine in the bladder, is paralysed, and thus allows the urine to run out as soon as it enters the bladder from the kidneys, which it is continually doing. Retention of urine may arise from something blocking the urethra, as a calculus or tumour, or more frequently from something pressing on the neck of the bladder, such as a pelvic tumour, or a backward displaced uterus, especially if it is pregnant. The woman is unable to empty her bladder, and the result is that it becomes distended to its utmost limit, and then fortunately an overflow begins and there is a continuous dribbling of urine just as in incontinence or with a fistula. If this did not happen the bladder would rupture, so it is very fortunate that it does occur.

To decide which of the three conditions is present the first thing to do is to palpate the abdomen. If one finds a large tense tumour extending as high or higher than the umbilicus, one may be pretty sure it is a case of retention. The tumour felt is probably the distended bladder, but it might be some other tumour. To make absolutely sure the next step is to pass a catheter. In some cases a pliable catheter will have to be used as the urethra may be distorted. The catheter must be aseptic, and before passing it cleanse the vulva very thoroughly, and be especially careful to wipe the meatus. If you see urine trickling from the meatus you can decide that it is not a fistula, so it must either be retention or incontinence. If it is a case of retention you will draw off a large quantity of urine when the catheter is passed. It is well to remember that the whole of the urine should not be drawn off at once, or very severe hæmorrhage may occur from the mucous membrane of the bladder when the pressure is removed. If the urine is very septic it should all be taken away, but 10 ozs. or so of boracic lotion should at once be injected into the bladder. The cause of the retention will now have to be sought for, and the treatment will consist in removing this cause.

In a case of incontinence the bladder will not be distended, and on examining the parts carefully urine will be seen to dribble from the urethra. On passing a catheter, the bladder will be found empty.

In a case of fistula the bladder will be empty and no urine coming from the meatus. If the opening is at all large it can easily be felt on making a vaginal examination. It will be found in the anterior vaginal wall a little below the cervix, and sometimes extends into the anterior lip of the cervix. One may be able to pass one or more fingers into the bladder. By using a Sims' speculum to draw back the perineum and posterior vaginal wall the fistula will be exposed to view. The bright red mucous membrane of the bladder will be seen protruding, and urine trickling from the opening. When the fistula is very small, it cannot be felt or even seen unless a coloured fluid is introduced into the bladder. Boiled milk is a very convenient fluid to use; a few ounces of which are injected, and a careful watch kept to see where it oozes through the wall. In rare cases it will be seen to ooze from the cervix which shows that the opening is between the bladder and the uterus, most frequently between the bladder and the cervical portion of the uterus.

*Treatment.*—In slight cases the opening may close of itself, but in most a surgical operation will be necessary. If the case occurs after delivery, *i.e.*, in two weeks or so, an immediate effect, operation cannot be done as the condition of the parts will be unhealthy and healing would not occur. Several weeks will have to be allowed to pass, and in the meantime the nursing of the patient will be difficult. You must endeavour to keep the parts as sweet and as dry as possible. Boracic vaginal douches should be given twice daily, and antiseptic dusting powders used freely. The draw-sheet and napkins will have to be changed very frequently, and, if possible, some form of receptacle should be arranged under the patient's hips to catch the urine. When the parts are in a fit state for operation it will be necessary to prepare the patient by thorough cleansing and douching several times for a few days. The bladder as well as the vagina should be douched out with boracic solution. To facilitate cleansing of the vulva the hair should be shaved off. The strong antiseptics like perchloride of mercury or carbolic acid should not be used as they are irritating to the mucous membrane of the bladder and poisoning may result from absorption.

The operation consists in rawing the edges of the fistula and stitching it up carefully so as to make the bladder watertight. The mucous membrane of the vagina alone is pared and the stitches are passed so as not to include the mucous membrane of the bladder. The latter is very vascular so if it is cut or a stitch passed through it severe hæmorrhage may occur into the bladder after the wound is closed. Silkworm gut makes a very convenient suture. When all the sutures are tied the bladder is filled with a coloured fluid to see if it is quite watertight. If there is any leakage more stitches have to be inserted. The stitches are left long to facilitate their removal. Some operators leave a retention catheter in the bladder to drain the urine out continually for a day or two, while others have a catheter passed every few hours, and allow the patient to make urine herself as soon as she is able. I prefer the latter plan, as there is less risk of cystitis being set up. The stitches should be removed in 10 days or so. If union has not taken place throughout, another operation will be necessary as soon as the parts are healthy. In very large

VESICO-VAGINAL FISTULA—*Continued.*

fistulæ, where the greater part of the base of the bladder has been destroyed, the tissues cannot be brought together, and then either the uterus or the cervix may be utilised to fill the gap, or in elderly women beyond the menopause, the vagina may be entirely closed. In cases due to malignant disease, nothing can be done in the way of closing the fistula. The only thing to be done then is to keep the patient as dry and clean as possible.

*Recto-vaginal Fistula.*—The opening may be above the perineal body, or that body may be partially or entirely torn through. When the perineum is entirely destroyed we have a condition which is known as a cloaca, like what naturally exists in birds. If the opening is of any size fæces, especially when fluid, will come through the vagina. If the opening is very small, only flatus may escape through it. This may cause great annoyance to the patient, as she has no control over it, and the flatus may come away with a gurgling or whistling sound at awkward moments.

*Treatment.*—If the bowels are kept very constipated the patient may not suffer very much inconvenience, but the least diarrhœa will cause her very great inconvenience, as she will have no control over the bowels. As soon as the parts are in a condition to admit of operation the bowels should be very thoroughly cleared and the parts cleansed by boracic douches. The fistula is treated in the same way as that of the bladder by rawing its edges and stitching it carefully, but the operation differs in that catgut stitches are put into the mucous membrane of the rectum and tied inside the bowel. The vaginal mucous membrane is then stitched, and if the perineum has been torn, as is usually the case, it is repaired by passing deep stitches through it after it has been carefully rawed. Care has to be taken to bring the ends of the sphincter muscle together. The bowels may be kept confined for some days, and then an enema of warm olive oil may be used to ensure that no hard masses pass, as these may cause tearing. The perineal stitches may be taken out in 8 or 10 days. It is well to remember that retention of urine is very common after any operation on the rectum or perineum, so that a catheter will probably require to be used for a day or two.

### Beyond the Seas.

### NURSING IN THE MELBOURNE EYE AND EAR HOSPITAL: BY THE MATRON.

It is not very often in ordinary hospital experience that it falls to the lot of those engaged in the nursing profession to see, at one time, five members of the same family as in-patients in the same hospital, all undergoing treatment for the same affliction, although in fever and infectious hospitals it is by no means so uncommon as it used to be.

The cases I allude to now, however, were five patients suffering from cataract of both eyes (congenital), and were treated in the Melbourne Eye and Ear Hospital—a mother and her four children.

The young woman was born in the country, or " bush " as it is called here, with congenital cataract, grew up, and married a bush labourer, without any idea that her disease, under efficient treatment, was quite curable; four children were born to this couple, each of them like their mother with cataract, but when a fifth child was born it was found to have taken after the father, and its eyes were quite perfect.

Hearing in the nearest township of the remarkable cures effected in the city, the parents resolved that as soon as the youngest child was old enough to be left with its father, the mother and four eldest children should endeavour to get to Melbourne and gain admission to the Eye Hospital.

They succeeded in their object. It was their first visit to a city, and they presented a most rare and pathetic sight, but those who saw them arrive were struck with the cleanliness and neatness of the children and of the mother herself. This was the more wonderful because she could barely see daylight, and the tidy appearance of the little ones was enough to make many a woman blush who possessed the full use of both eyes.

The two girls had their hair cropped quite short like the boys, and the woman herself had her own cropped also. She explained that she found this the easiest way of keeping it tidy, and no doubt the husband did the barbering.

She also told me that these little children, who were practically almost blind, used when at home to go off for miles into the bush, playing their games and, almost with the instinct or scent of animals, speedily finding their way back. Never once did they get lost, which, here with children and even with grown-up people, is a matter of all too frequent occurrence. In most parts of the country there is no beaten track so that unless bearings are taken, such as " ringing " the lower part of trees or some such precautions, it is easy to get " bushed " as it is called.

Lost children are generally sought for by black trackers and by trained bloodhounds; sometimes they are never found at all, often they are in a dying condition from their dreadful position and privations, whilst on the other hand, some appear little the worse for their experiences and have lived by eating gum off the trees; the fondness for this food shown by some people has gained for Victorians the soubriquet of " Gum-suckers."

The treatment in the hospital of these patients was the one usually adopted in the case of young children or people under 30. It is a very slow process, and calls for great patience and perseverance. Sometimes it takes six or nine months to complete both eyes.

However, in due time all were successfully given the most blessed gift of sight, and discharged as " cured." The mother's joy was intense as she prepared to take the little ones back to their father waiting in the bush home, and her praise of the hospital could scarcely find expression. It seemed almost impossible that this family who had arrived groping along and feeling their way as they went, or led to the wards by the nurses, could be the same who now walked out fearlessly and brightly, able as they looked round to see all perfectly well. Surely, no greater benefit could have been conferred on an afflicted family!

### Presentations.

Kensington Infirmary.—Miss E. L. Chippendall, on resigning her post of night superintendent at Kensington Infirmary, has been presented with a handsome travelling rug, medical dictionary, and a silver-mounted umbrella, from some of the nursing staff. Miss Chippendall has accepted the post of superintendent nurse at Dorking Union Infirmary.

### To Nurses.

**We invite contributions from any of our readers, and shall be glad to pay for " Notes on News from the Nursing World," or for articles describing nursing experiences, or dealing with any nursing question from an original point of view. The minimum payment for contributions is 5s., but we welcome interesting contributions of a column, or a page, in length. It may be added that notices of appointments, entertainments, presentations, and deaths are not paid for, but that we are always glad to receive them. All rejected manuscripts are returned in due course, and all payments for manuscripts used are made as early as possible after the beginning of each quarter.**

### Papers for Private Nurses.

BY A SISTER OF A LONDON HOSPITAL.

(*Continued from page* 39.)

### Night Duty.

A very full share of night duty falls to the lot of the private nurse, chiefly because it is so much more satisfactory when single-handed to take this portion of the work entirely into her own hands. The friends often suggest taking turns—nurse sitting up one night, and they the next—but this never answers. Whenever I have been alone at a private case, I have always taken night duty from the first night until the patient was convalescent, and any help I have asked from friends or servants has been during the day time while I rested. It was always my first object to get the friends out of the sick-room, and I generally found the argument that I wanted them to be rested in order that they could take my place next day, one that materially helped me in securing my aim, and thus gaining quiet and freedom from "fussiness" for the patient. A nurse's hours of rest at a bad private case when single-handed are always short enough; but my experience has been that during the day the friends tried to do without me as long as possible. When the patient could be left to the friends for some hours, I have generally gone on duty between 9 and 10 p.m., and then remained on till after the doctor's visit next morning, which has generally been by or before noon. After this, if I could get a walk for an hour I always did so, and then, seeing first that my patient was comfortably settled, I had some lunch and went to bed. Sometimes of course it is necessary to be called during the resting time, possibly to give an injection, or a nutrient, or to do a four-hourly dressing, but in other cases the friends often manage very well. They do not become frightened in the daylight.

### The Most Convenient Hours.

My first duty on returning at evening would be to wash and settle the patient for the night—and though to a nurse fresh from hospital 9.30 or 10 o'clock will seem very late, a private patient will often call it early. Patients have a way of watching the passing hours, and to those who rarely retire till midnight or later the dread of such a long night will make them anxious if one darkens the room early. In a middle-class family where there are not many servants, these hours prevent the necessity for preparing any separate meals—an arrangement which always gives much relief to the friends, who, when obliged to call in a nurse for night duty, wonder how this matter is arranged. If she goes on duty about nine o'clock, the nurse's first meal can be kept from the family dinner or supper, according to the habits of the household, with the addition of some tea or coffee. Many people will kindly bring a cup of tea when they call nurse. I have often received much consideration in these matters, far more thau I ever expected. Then having had a reasonably substantial meal before going on duty, I have found tea and some bread-and-butter or biscuits as much as I wanted till breakfast time, which I have either had with the family, or as soon as someone could come to relieve me. The morning's duties in the sick-room, the doctor's visit, and a walk bring the time for the midday dinner or lunch as the case may be, so that all trouble of separate meals is saved.

### Compensation for Hard Work.

To go to bed at 2 p.m. and to get up before 8 p.m., having been called up once or twice in between, is not, of course, sufficient rest, especially if continued for a week or two, but in the early days of a private case, unless there be two nurses, it is often much more than one can get. All private nurses of experience know the weariness of it—a very different weariness to that of hospital night duty, with its active life and regular hours off duty. But to be alone with a patient struggling through a severe illness, or after a serious operation, hard work though it may be, has its compensations to the true nurse, and not to anyone does she care to give up the duty that she is best able to perform. To be "skilled in comfort's art" is her highest aim, and her opportunities for becoming so are many in the long quiet hours.

### The Question of Fire and Light.

The fire and lighting of the sick room need most careful management, but a nurse should never be prevailed upon to do without a light, although friends and patients sometimes will tell her that she must not have one. A little tact in her answers to these objections will smooth away the difficulty. I have always said I could not promise to keep awake, nor observe the changes in the patient's condition if I had to sit in the dark, reasons which invariably appeal to the patient's friends, while careful shading of the light will satisfy him. I use either candles or a small lamp—never gas or electric light. The candle I place on a small table, not too near the bedside, with a screen between it and the patient, or at the foot of the bed with a rug or counterpane spread over the foot of the bedstead, and a large open book around the candle or small lamp, and this will greatly brighten the light for the nurse's own use. The fire can be prevented from flaring and making a brilliant light by using small coal and then sprinkling ashes on top. A thin layer of ash will keep down the flames, and if the fire be gently raked from the bottom with a wooden stick, there need be no noise of poking. It is a good plan to have coal put up in small brown paper bags—brown paper will not flare up—and any noise occasioned by using a shovel will be prevented. A nurse should never forget to take advantage of the patient being awake, to attend to the fire and light.

### Small Meals and Occupation.

Nor should she take her small meal in the patient's room if she can possibly avoid it. It is better to have a little table outside the door if she cannot go into an adjoining room. It is almost impossible to help making a little noise, but by choosing a time when the patient is dozing he need not be disturbed, and she will easily hear any sound inside the room for her ears will become accustomed to the least change during the night's long quiet hours. She will often find it most difficult to keep awake, even though she may know that an important symptom may be missed by her falling asleep, or a movement that may be bad for the patient pass unnoticed. A very wise plan is to have plenty of occupation, and of varied kind, a book or two of quite different subjects, sewing and fancy work that can be done quietly, and letters to write. The latter I always found helped me to keep awake. The temptation to sit in a comfortable chair and doze is a very great one—especially when the nurse has had but little sleep during the day, so that she feels utterly tired out when the "small hours" are reached. It needs all her devotion and conscientiousness to be faithful in this matter. But to be trusted absolutely with the lives of her fellow creatures is a great and noble task, well worth the effort and self-denial it needs, and the lines that have often soothed a weary patient, have a meaning no less for nurses—

"God gives His angels charge of those who sleep
But He Himself watches with those who wake."

(*To be continued.*)

### Wanted, a Probationer: Also a Cook.

BY A MATRON.

The day on which I received the official announcement of my appointment as matron to the Willinghurst Cottage Hospital was a red-letter day in my life. I had served three years' probation in a London general hospital, and another three partly as night superintendent at one of the best special hospitals in the metropolis, and partly as ward sister in a large provincial infirmary. Happy as on the whole I had been in the work of my various posts, I had found much to complain of in the ward and domestic arrangements of these otherwise well-regulated hospitals which entailed endless discomfort on the nursing staff, and to which as a powerless sister or probationer I had to submit. How any matron or housekeeper could put up with the ordinary hospital cook I could not understand. Something (I was not quite sure what) ought to be done, and someone (I was not sure who) ought to be called to account. Then again the probationers, who as ward sister I had to train. Why on earth did the matron, who was otherwise a sensible and clever woman, allow such idiots to enter the hospital? It was well known that the applications were numerous, so why were these particular women chosen, when numbers of healthy, intelligent, well-educated applicants were longing to become nurses? Alas! I little thought how soon from my own experience I should be able to answer these questions.

### A Free Hand for Reform.

Now that I had obtained the object of my ambition and become a matron, I determined to spare no pains in showing the world what a happy, comfortable place a hospital could be. Of course it would require much care, trouble, and watchfulness; indeed it was to the want of these qualities on the part of responsible officials that the many shortcomings from which I had suffered seemed to me due. But I was young, strong, and energetic, thoroughly devoted to every department of my work, and determined not to spare myself in my efforts to make all around me happy and comfortable. In my ignorance and inexperience I fondly thought that nothing else was needed for the working of a hospital that should not only be a model temple of the healing art but a real home to the workers. No better field I thought could have been found for the carrying out of my theories than the hospital to which I had the good fortune to be appointed. Small enough to be worked on the "cottage" principle, and large enough to support a staff of two nurses, the same number of probationers, and three servants, no resident medical officer, no ladies' committee, and no secretary within call. The general committee meeting only once a month, the working of the hospital was nearly entirely in my hands, and in consequence an unusual amount of responsibility rested on my shoulders. But I was not afraid; besides, it gave me a freer hand for reform.

### Answers to the Advertisements

I paid my future home a visit of a few hours before starting for the holiday that was to prepare me for labours in fresh fields. I was quite glad to find that it was much as other hospitals—no worse, but certainly no better; and I experienced a distinct throb of joy on hearing that the senior probationer would shortly be leaving on completion of her term, and "I am sorry to say," continued my predecessor, "that the cook has just given warning, and her time will expire two days after your arrival. I have tried to arrange for her to stay a little longer, but she can only remain a few days beyond the time she is due to leave." "And quite enough, too," I thought, remembering the very plain lunch I had just eaten but not enjoyed. It seemed a stroke of good luck that I should be able to inaugurate the new order of things without delay. I inserted two advertisements in The Hospital, timed so that I should find the answers awaiting me on my arrival at Willinghurst. There they were: quite a large pile. There certainly would be no difficulty in filling the vacancies, both nursing and domestic, and as I had especially requested applicants to give full particulars, two or three days would amply suffice to make a few final arrangements. After an early dinner—nearly the last of its kind I thought with joy I need ever eat—I set to work to open the letters. Tea-time found me with an aching head and reeling brain, surrounded by documents of which I could not always trace the writer, and the floor peppered over with loose stamps fallen from envelopes, I could not always remember which. At least twenty writers told me they were "very fond of nursing" and nothing else, whilst a few more sent me four pages of family history. A post-card from someone who would like to know by return of post if the engagement could be kept open a few weeks as she wished to go to a wedding, and attend some garden parties before settling down as a nurse. A few more, mostly signed with initials, asking for full particulars, and giving none. One required a ménu of the meals and plan of the hospital, another a pattern of the cap: she wished before applying to make sure it would become her. A girl of 16, whose parents had consented to her "going forward as a hospital nurse," and a woman of 45, who felt sure her devotion to the work, of which as yet she knew nothing, would atone for her superfluous years, came next. Then a young man who applied for "a particular lady friend," and a father who felt sure his daughter had only to be seen to be engaged; "she looked every inch the nurse," and would come to see me on receipt of return fare (she lived 200 miles off). A long letter dated from a country vicarage, whose writer trusted I would excuse a mother's anxiety in asking if I saw to the airing of the nurses' underclothes myself. A small handwriting in red ink caught my eye. It ran: "I am a lady (underlined), but in consequence of temporary financial difficulties am obliged at present to ask for a salary. When, however, I am in a better pecuniary position, I should amply endow any institution that engaged my services; indeed, should have much pleasure in doing so." Another was "devoted to the study of physiology and anatomy," and "anxious to be placed under circumstances that necessitated the study of these sciences." Several who had failed in London hospitals felt sure they would succeed in a country one. Delicate girls who in consequence wished for country air; dwarfs under 5 feet, and a giantess of 6 feet. One confessed to deafness, another had an impediment in her speech. A cripple, whose lameness prevented her from going to domestic service, but who the kind lady who wrote on her behalf thought especially fitted for nursing work. Many enclosed photos with return envelopes too small to contain them, and another would, if engaged, require a much higher salary than the one offered. One and all were quite sure they were the one woman suitable to be probationer to the Willinghurst Cottage Hospital.

### The Last Application.

I was about to give it up and go to bed when I discovered one more letter still unopened. Here surely was all that I wanted, and I felt that my ideal probationer was at last found. I answered at once. The enclosed envelope was stamped but not directed, so I turned to the heading of the letter for the address. There was none. Name Smith, postmark London. That night I dreamt of probationers, tall pros., short pros., thin pros., fat pros. They crowded round my bed clamouring to be engaged under pain of ——. A knocking at my door woke me. The housemaid with my hot water and a message. "Please matron, cook wishes me to tell you she has had a letter and would like to leave to-morrow. She is sorry to inconvenience you." "Oh, not at all," I interrupted, and then it flashed across me that among all those applicants there was not one cook. A year afterwards I, a wiser and humbler woman, ate, with gratitude, dinners prepared by that very cook, whose services I had with thankfulness retained, and snubbed a probationer who disparaged her cooking.

### Nursing in the Burgher Camp, Kimberley.

BY A SISTER.

Our nursing began soon after we left England, for we had several pneumonia cases on board, and divided duty between us. When we arrived in Capetown we had to wait several days, and were able to enjoy the scenery. We also had to buy food and fruit for our journey in the train up country, not to mention sun bonnets, for none of the nurses wear proper uniforms so far. Washing is almost an impossibility, so we look a very motley gathering. All the sisters except three went to the Orange River Colony. Another and myself arrived here on a Sunday afternoon; we saw a good deal of the country on our way. The Karoo part impressed me very much with its hundreds of miles of dried earth, stunted bushes, and mountains on either side. In the hospital camp we found plenty to do under our pleasant matron. The sanitary arrangements are beyond description; but there were five nurses short when we came, so that now we hope to be able to organise matters. This will take a good deal of tact and hard work.

### Dutch Probationers.

I am on night duty with six Dutch probationers. Some of them have only been nursing a month: they are pleasant and obliging girls, but have not yet learned that they must not sleep on night duty, and must be careful to obey orders. Their English is very imperfect, and my Dutch worse, so that it is not easy to get along. When I am away sponging a typhoid patient or washing in other wards, I do not know in the least whether one of these probationers will be carrying out orders or not, but this does not bear thinking about. I simply go from case to case and keep as watchful an eye as possible over the nursing. I found the other night a poor boy having brandy hourly instead of every six hours; but the flaccidity of these people is simply marvellous. They seem to take correction pleasantly. My pro.'s go on duty in the most varied dresses in point of colour and material. At present that matters little so long as they will learn.

### The Divisions of the Camp.

To me the camp seems large. It contains more than 3,700 people. It is a wonderful sight, and the points of the tents are decidedly pretty against the brilliant sky at sunset or in the bright moonlight. The camp is divided into Hospital, Rebel, and Refugee. The hospital enclosure is comparatively small and fenced round with barbed wire. We are always locked in. On two sides of us is the rebel camp: this also is carefully locked. My tent is quite close to them. I can hear everything they say, but as they talk Dutch I am not any the wiser. The temperature of my tent is 92°, and with the noise the days are not restful. The refugee camp is on the other two sides of us and open to the road.

### The only Englishwoman on Duty.

I do not think the English sisters have anything to fear from the Boers. Their women always welcome us and give us a cheery good morning. I find the nights rather gruesome though, for I am the only Englishwoman on duty. My shins are suffering from lack of skin taken off by the tent ropes. Still other things are not so bad. The Boers here are not so dirty and lazy as we used to suppose at home, and are more warm-hearted than I expected. They are amusing, and sing hymns at intervals all day, from the time they get up at 5 A.M. The tune only is familiar, of course. It is a pity that the women hide their sick: they will not let them come into hospital if they can help it. We have a good many diphtheria cases, but nothing will persuade them to have tracheotomy done. At present I have three patients. The form of enteric is very curious, with a temperature only lasting ten days or so. Perhaps I shall find longer cases later on.

### Furniture of the Tent.

My tent is my only resting place when I am off duty. I have a table, a washstand, a stretcher-bed, and a bath; two blankets on the floor for a carpet, a mackintosh to cover up the bed when it rains. Towels and other luxuries we find ourselves. I have my deck-chair, and a cushion which was given me before I left England. Papers would be most acceptable. They should be addressed, "The Nurses," Burgher Camp Hospital, Kimberley.

### My Masseuse.

With sleeves tucked up and smile sedate,
She enters as the clock strikes eight,
She's never half a minute late,
My Masseuse!

She smears my face from brow to chin
With vaseline, grease, or lanoline;
She says they're splendid for the skin,
My Masseuse!

She says I need not fret nor fear,
The wrinkles all will disappear;
My beauty will return—next year!
My Masseuse!

She exercises all my toes,
To every little joint she goes,
The while she listens to my woes,
My Masseuse!

She works away at every limb,
She says I must get nice and slim;
She knows that is my latest whim,
My Masseuse!

I'm rather frightened when she flings
My arms about like flapping wings;
She's very calm about such things,
My Masseuse!

She prods me with the softest thumb,
She beats me like a little drum,
And all the while I must sing dumb,
My Masseuse!

She turns me o'er like a sack;
With tap and slap and smack and whack
She well manipulates my back,
My Masseuse!

Then lo! a shower of finger-tips
Like rain descends in flicks and flips;
And this is how my spine she whips,
My Masseuse!

Then having pinched, and rubbed, and such,
She strokes me down with velvet touch:
Of this I cannot have too much,
My Masseuse!

But just as I'm beginning to
Enjoy myself off she must go.
Alas! in everything 'tis so,
My Masseuse!

She goes to soothe another's woes,
To lubricate some other nose,
To exercise ten other toes,
My Masseuse!

To-morrow, with her air sedate,
She'll enter as the clock strikes eight;
She's never half a minute late,
My Masseuse!

### Everybody's Opinion.

[Correspondence on all subjects is invited, but we cannot in any way be responsible for the opinions expressed by our correspondents. No communication can be entertained if the name and address of the correspondent are not given as a guarantee of good faith, but not necessarily for publication. All correspondents should write on one side of the paper only.]

### WAITING ON THE KING'S GUESTS.

"Policy-holder 6855" writes: I have been reading of the difficulty of getting people to wait on the King's guests at the dinner to be given by him to the poor of London. May I suggest the help of nurses for waiting? I think many nurses engaged in private nursing and at liberty would gladly volunteer their services for a few hours. I should be most willing to do so myself if in London at the time and able to leave my patient, and if not at a case I could give the whole of the day. I feel sure that the poor would appreciate being waited upon by nurses.

### A SCHEME FOR AFFILIATED HOSPITAL NURSING.

"Another Matron" writes: I was pleased to see the letter in your issue of April 12th from "A Provincial Matron." I have always thought it hard lines and very discouraging for those matrons and nurses who try to do good work in the numerous provincial hospitals to be continually reminded of their inferiority by these city sisters: many of them have never investigated for themselves whether these things are true or not, or whether the majority do not keep abreast like themselves with modern ideas. Numbers are not everything; quality rather than quantity should be our aim. I have had fifteen years' experience in nursing; I have worked in three different hospitals, two of them city hospitals and one in the provinces. I may add that the work done in the latter institution was quite equal to what I saw in either of the other two, both by doctors and nurses. In some respects the nurses are better trained; they have a larger experience of practical work where there are no students. We had no lack of probationers, and they had no trouble in finding work on receipt of their certificates at the end of their three years' training.

### THE CASE AGAINST HOSPITAL NURSES.

"E. S. B." writes: I am sure the Lady Superintendent of the Birmingham and Midland Counties Sanatorium will have the thanks of all nurses for her defence to the charges made against them in the *Nineteenth Century*. I think that anyone who makes such sweeping remarks must either have been most unfortunate and unusual in her experience of nurses, or that she cannot know anything at all of the true life of a nurse. Quite lately I was reading an account of a meeting of the Board of Guardians of a town in Essex, at which one of the lady guardians who had been delegated to attend the Poor Law Conference in London said that nurses who were trained in the ordinary hospitals would not settle down to infirmary nursing as they looked for too much "pleasure, amusement, and dissipation." I am quite sure that this is not true of real nurses. I consider that all honour is due to those who spend their lives looking after the chronics. They have not even the hope of recovery to inspire their efforts; but there are many doing their best for these poor sufferers, and anyone doing this is quite right, and it is a duty to herself as well as to her patients to embrace every opportunity that may present itself of obtaining pleasure, not dissipation. Bearing in mind the usual class in Poor Law infirmaries a nurse can hardly be expected to keep up her spirits and wear a smiling face—no trifling matter for the patients—if she does not mix with her friends at intervals. A guardian should hardly need to be reminded of this fact.

"G. F.", Milverton, Somerset, writes: Reference has been made in your columns to Miss Johnston's article, "The Case Against Hospital Nurses." Having for the last 14 weeks had experience of the value of the hospital nurse in her capacity as nurse in a private family I think it only right to testify to the fact. First, with regard to her nursing powers and presence generally in the sick-room, I need only say that she is of such comfort to the patient that I trust she may be able to stay as long as the services of a nurse are required. Secondly, with regard to her presence in the house outside the sick-room, I should like to quote two of the rules of the Taunton and Somerset Hospital, to which this nurse belongs, as they have been most faithfully adhered to on her part, and therefore instead of her being any inconvenience to the household during these many weeks she has, on the contrary, been a most pleasant and helpful guest. Rule I.: "Nurses in charge of private cases . . . are earnestly requested to bear in mind that sickness of necessity brings trouble and anxiety, which are liable to be increased by the arrival of a stranger. They are therefore urged to cause as little inconvenience as possible to the household, and to do their utmost to gain the confidence of their employers, thus becoming a source of help and comfort to all concerned. III.: They shall adapt themselves as far as possible to the customs of the family in which they are on duty, with reference to the time and place of meals, and in all other respects. They shall arrange as to their proper hours for sleep and exercise, with due regard to the welfare of their patient, and the convenience of the household in which their attendance is required." I am very glad from my present experience to be able to bear testimony to the worth of the nurses sent out from the hospital of my own county town, and I hear of others in this neighbourhood who have had a similar happy experience of them.

### HOSPITAL REFORM IN FRANCE.

"A. G." writes: The twentieth century is somewhat apt to plume itself upon its superiority in hygiene, decency and cleanliness to its predecessors, but it is a certainty that our descendants will find us quite as worthy of condemnation on these points, in some ways, as we do our ancestors. There is a wide field indeed for such reforms in our hospitals, and your article on "Hospital Reform in France" points the way towards many improvements. No word is, however, said upon a most important, a most vital subject, that is, the treatment of the living machinery, by means of which the work is carried on; walls are to be kept clean, efficient isolation practised, ventilation is to be perfect, and so forth, but nothing is said as regards the nurses, nor of the reforms which are so urgently needed in their position. I believe that in America—and alas! for the frequency with which America teaches us lessons which we should rather be expounding to her—female nurses are not allowed to give the bed-pan to male patients. I am convinced that our descendants will look back with horror and disgust unspeakable to this our time when young women of delicacy are compelled to perform this office, not only to patients whose extreme weakness or illness renders it less objectionable, but also to robust men in hospital for surgical troubles quite unaffecting their general health. There should be in all hospitals male attendants (who need not be in any respect trained nurses) employed to give the bed-pan to all male patients, excepting in the cases where some special treatment may be in progress. Absolutely no advantage is gained by enforcing this duty upon girls, and so far as it affects them at all must tend to coarsen them. Even worse is a custom which is, I hope, confined to a certain hospital: I allude to the sending young probationers to the lavatories used by convalescent men to pull the plugs, etc. This is a barbarous idea, a needless humiliation to the girls, and a duty which should be performed by a man. The hours of work are also usually far too long for nurses, the continual occurrence of flat-foot amongst them being one amongst many evidences of this. The work is, under any conditions, very arduous and fatiguing, and the amount of suffering patiently and silently endured by nurses would indeed startle the uninitiated could it be made clearly known. More time for rest and reasonable recreation is imperatively needed for nurses in most hospitals. The girls are often so fatigued when off duty that they are fit for little else than rest in bed. Miss Gardner's article, "The Case against Hospital Nurses," is excellent in many ways, but I think she fails to see that there is a substratum of truth in Miss M. T. Johnston's article in the *Nineteenth Century*. Hundreds of women pass through their hospital life and emerge neither coarse, callous, nor selfish; but it is true as truth itself that long hours, over-fatigue, and petty tyranny do exist in many hospitals, and such reforms are needed as to make it sure, not merely that women shall by chance pass through the mill uninjured, but that hospital life shall never tend towards the creation of these faults. There are excellent matrons and good and sympathetic sisters in numbers in the profession (I only wish I might mention a few names), but they are often powerless for lack of means to do that which they would dearly like to do in amelioration of their nurses' lot. The hard grind, the hurry in which everything must be carried out at many hospitals does tend towards a bluntness of feeling, and we should rather note with satisfaction the number of nurses who pass unscathed through a fierce trial than blame those who succumb. The undermanning or rather no-manning of the staff from motives of economy is the main source of overwork and too long hours. On this point I would beg the authorities of our hospitals to consider that the nurses are always at least as well worthy of consideration as the patients, numbers of whom, especially amongst the men, are suffering the consequences of their own wrong-doing. Surely a young woman who has possibly left home and friends to lead a life of toil and of self-abnegation is as worthy of care as a drunken bricklayer who has been hurt in a fight; surely also, a nurse should be as sedulously protected from overwork as a factory hand or a dressmaker's apprentice. What can be said too, of a hospital, and there is one, where nurses are not allowed to sit down while on duty except at night?

### SLEEPING IN A PATIENT'S BEDROOM.

"Medicus" writes: As a member of the medical profession, and of the opposite sex, may I be permitted to express my views on the subject of nurses sleeping in the patient's room, etc.? Such a practice is, *ipso facto*, highly undesirable, but in the circumstances mentioned by your correspondent hardly to be avoided. But after all such considerations are more in the domain of Mrs. Grundy than of the nursing profession. Not that I have aught to say against Mrs. Grundy, whom I look upon with the utmost regard when not overdone. But surely this is straining at a gnat and swallowing a camel, for what seems to me of infinitely greater importance is the fact that any woman should be permitted to pass a catheter on a man, except under the most exceptional circumstances. I have held a house appointment at a large London hospital and am now attached to a large military hospital. All I can say is that the mere idea of a nurse doing such a thing at either of these institutions would cause a feeling of utter disgust, and very rightly too. The practice is one that ought never to be allowed, and I am quite sure a way out of the difficulty could be found if both the medical man and the nurse were nice-minded enough! I have seen a good many cases of hemiplegia, some with incontinence and others without, but I cannot recollect any where it was necessary to pass a catheter every three or four hours. If so, surely continuous drainage could easily have been effected, which is certainly less dangerous than the frequent passing of a catheter. My experience in private practice is limited, but I should certainly be extremely sorry to ask any woman to pass a catheter on a man. In the Army a sister is not allowed to even dress a hernia case, and I think quite rightly too. I may say I have a sister a nurse and were she deputed to a "catheter case" I hope and trust she would refuse to take it. It is absolutely indecent. I heard a nurse described as a "sexless being"; that is a gross libel on the average nurse. There is not the slightest reason why a woman's feelings should be utterly outraged for the sake of any patient living, hemiplegic or otherwise. Women have it in their own hands and they ought to refuse to do what is essentially, and only, a man's work. Just ask a doctor how he would like his wife or daughter to do such a thing and see how the boot fits then. I fancy it will pinch a bit. Surely a nurse is entitled to just as much respect and consideration as any other woman. I am glad to read in your excellent periodical that there are still people who look upon a nurse as a woman with a shred of modesty and decency about her. I am quite sure nurses would earn far more respect by being a bit more "squeamish" than by their proneness nowadays to think they can do everything a man can. Certainly there is no art in passing a catheter, or very little, and I do not suppose that the staff at Macclesfield doubted the lady doctor had the skill to do a male doctor's work. They rightly and absolutely refused to allow a woman's feelings to be outraged at their place, even if she were willing. And I think this pretty clearly indicates the opinion of all decent-minded men who possess sisters, wives, or daughters. I trust that you will forgive my taking up so much valuable space, but I feel the subject demands it, owing to the increase of this utterly degrading practice.

### MALE NURSES IN GENERAL HOSPITALS AND PRIVATE WORK.

"William Gutteridge, Superintendent Male Nurses' Association, 23 York Place," writes: In your issue of April 12th there is an article with reference to the above subject. It is said in your "Notes on Nursing News" "that the present difficulty is to obtain a trained male nurse who is nearly as efficient as an ordinary probationer at the end of her second year." Such a comment is most unfair to the many fully-trained and capable male nurses who are being continually supplied to the public at the present time. The case mentioned in your remarks where a male nurse was engaged and through bad nursing the patient became rapidly worse, might apply equally well to a woman nurse, but to believe that such conduct is common, or that good and qualified nurses of either sex are guilty of treating their patients in that manner, is undoubtedly very misleading, and ought not to go unchallenged. The public have a good deal to thank themselves for in such matters. They advertise and reply to advertisements by so-called male nurses, when nine out of ten of such men are not entitled to call themselves by any such name. Their experience of nursing probably amounts to attending on some invalid in whose service they chanced to be in when the patient was taken ill. The fully qualified male nurse who has been trained in such institutions as the National Hospital, the Borough Asylum, Derby; the Berrywood Asylum, Northampton; and several other excellent training schools for men, where three years of real hard work and study must be gone through, and a strict examination passed, before the certificate of the Medico-Psychological Association is granted, is capable of nursing any case of illness with the best female nurse from our leading hospitals, as many doctors and female nurses could testify. Your contributor wishes to know whether it would not be better to employ male nurses in the male wards of hospitals. Such an innovation would not only be right, but sensible and proper, not only in the wards of hospitals, but in private houses as well. There are numerous cases in hospitals and private work which are not suitable for female nurses; in fact, there should be some rule between doctors and superintendents of nursing institutions in refusing to allow female nurses to attend male patients for catheter, massage, and other diseases which it is not necessary to mention here. Certainly a patient is at liberty to have whom he likes, even if a doctor advised a male nurse; but, as your contributor remarks, if a woman nurse refused to attend such cases what is becoming a growing evil would be checked. It is a great pity that one ward in each of our hospitals cannot be given up to patients nursed entirely by male nurses. A sister could superintend the work until a good man was capable of taking entire charge. It is possible for a man to do any part of nursing equally as well as a woman—in some cases with greater success—so the experiment could not be other than successful. A strict training should be compulsory, and no one given a certificate unless fully entitled to it. An arrangement like this would allow of all unruly and vicious patients in the hospital to be nursed by men, and a good deal of unpleasantness avoided. At the same time, if great care were exercised in selecting probationers, so that none but the right class of men were employed, there would be no danger of flooding the public with an inferior class of male nurses for private work. I am entirely in sympathy with your correspondent's remarks, but she does not seem to realise that there is an institution for supplying male nurses to the public. I have the honour to superintend such an institution, and I am proud to say that we are continually supplying good men for private work, not for cases "where little more than a valet is needed," but for surgical, catheter, mental, and medical cases. There are plenty of good male nurses, but they are not to be found by advertising in the daily newspaper; when these means are employed, it is very rarely that the proper fee is given, consequently only an inferior class of men is obtainable; a good man can always command the usual nursing fees, and by joining such an institution as this—carried on under the co-operative system—he receives good remuneration for his services, without the trouble and anxiety of finding work. It is the man who has had neither training nor experience in institutions who is doing so much harm to male nursing. With us it is necessary to hold a certificate of training as well as private recommendations from the different medical men under whom the man has been trained before he is registered on the books. It is easy for the public to remedy the matter by refusing to employ an untrained man. If this be done a hard-earned favour will be conferred on hundreds of properly-trained and qualified male nurses.

### Novelties for Nurses.

By Our Shopping Correspondent.

### UNSHRINKABLE PURE WOOL UNDERWEAR.

I am pleased to draw the attention of my readers to the "Griffin" brand of woollen under-garments. The quality is excellent and the finish good, and they are most soft and comfortable in use. Would-be purchasers must not omit to ask for the "Griffin" brand garments of their drapers, but should they meet with any difficulty Messrs. F. and W. E. White, Limited, of Loughborough, will supply them. The makers of the "Griffin" brand underwear are so confident of the character of their productions that they offer to exchange any garment should it become too small through shrinkage in the laundry.

### AN ADMIRABLE BOOT-TREE.

Boots and shoes cannot be kept either tidy or shapely without the use of a shoe-tree. But boot-trees are not nearly as much used as they should be, as there are so few which are entirely satisfactory. The London Shoe Company, of Queen Victoria Street and Bond Street, are now selling a really admirable tree. It is simple, well made, and durable. The illustrations show the method adopted, which is ingenious, and the mechanism cannot get out of order in ordinary use.

### Wants and Workers.

Would any nurse like The Hospital the Monday after publication? If so, please send 13 stamped and directed envelopes, M., District Nurses' Home, Cannon Street, Taunton. Post-card first.

### Appointments.

Brymbo District Jubilee Nursing Association.—Miss Read has been appointed Queen's Nurse. She was trained at Chester Infirmary.

Burnley Union Infirmary.—Miss Elizabeth Robinson has been appointed charge nurse. She was trained for three years at Chorlton Union Hospital, and possesses the L.O.S. certificate.

City Hospital for Infectious Diseases, Newcastle-on-Tyne.—Miss Julia Swallow has been appointed night superintendent. She was trained for three years at Northampton General Infirmary, and has since been sister of the men's ward in the same institution. She has done private nursing in Manchester, and has been night superintendent at the Borough Hospital, Croydon. Miss Swallow took a full course in midwifery at the Royal Maternity Hospital, Edinburgh.

Cottage Hospital, Harrogate.—Miss Bertha Taylor has been appointed staff nurse. She was trained for three years at the Royal Infirmary, Bradford.

Dewsbury Workhouse Infirmary.—Miss Janie E. Woodworth has been appointed charge nurse. She was trained at Salford Union Infirmary.

Dorking Union Infirmary.—Miss E. Chippendall has been appointed superintendent nurse. She was trained for three years at Kensington Infirmary and was subsequently charge nurse at the South-Western and Brook Hospitals, did private nursing on the Putney and Kensington Nurses' Associations, and for the last two years has been night superintendent at Kensington Infirmary. Miss Chippendall holds the L.O.S. certificate.

Mansfield Accident Hospital.—Miss Lily Baillie has been appointed staff nurse. She was trained at Wirral Hospital for Sick Children and the Samaritan Free Hospital, London, and has since been staff nurse at the Children's Hospital, Nottingham, and on the private staff of the General Hospital, Newark.

National Maternity Hospital, Dublin.—Miss Mary J. A. Hannan has been appointed lady superintendent. She was trained at St. Vincent's Hospital, Dublin, where she has since been staff nurse and night superintendent. She has also had fever training at the Fever Hospital, Cork Street, Dublin.

Norwich Isolation Hospital.—Miss Lucy Sharrock has been appointed assistant matron. She was trained for three years at the Royal Albert Edward Infirmary, Wigan, and was afterwards charge nurse at Brook Hospital, Shooter's Hill; sister at the Rotherham General Hospital, and sister at the National Orthopædic Hospital, Great Portland Street, London.

Poplar and Stepney Sick Asylum, Bromley.—Miss Maude Hendy and Miss Florence Mary Moles have been appointed sisters. Miss Hendy was trained at Crumpsall Infirmary, Manchester, and has since been sister at the Royal Infirmary, Wigan, and the Monmouth and County Hospital. Miss Moles was trained at Guy's Hospital, London, where she has since been staff nurse. She has also been charge nurse at Park Hospital, Hither Green, and sister at Fulham Infirmary.

Western Fever Hospital, Fulham.—Miss Emma Julia Howard has been appointed charge nurse. She was trained at Mile End Infirmary, London.

Whitworth Hospital, Darley Dale, Matlock.—Miss Ethel Fisher has been appointed staff nurse. She was trained at Smallwood Hospital, Redditch, and has since, for two years, done private nursing in connection with the Malvern Nurses' Home.

### Death in Our Ranks.

We are asked to announce the death of Miss Mary Anne Johns, Queen's Nurse since July 1900, at Gloucester. She was trained at the General Infirmary, Gloucester.

### Echoes from the Outside World.

### Movements of Royalty.

The Queen returned from Denmark to London on Tuesday. She has been absent from England since March 26th, and her reception on her arrival at Charing Cross Station was very enthusiastic. The King and the Prince and Princess of Wales met her, and as the carriages containing the members of the Royal Family drove away there was a popular demonstration from the people outside. Her Majesty, who appeared to be in excellent health and spirits, spoke a few words to the Danish Minister and his wife, who were amongst the company on the platform.

Princess Louise, Duchess of Argyll, presided at an entertainment of the Royal Drawing Society at Queen Anne's Gate, on Friday last week. All the tickets and the pictures were sold for the benefit of the Soldiers' and Sailors' Families Association, and the competition was a most interesting one. A bouquet of beautiful roses was offered to the Princess by a little girl of nine, Eileen Hood, who is the winner of the Princess' gold star given every year. She is the youngest child who has ever earned the distinction, and when she presented the flowers the Princess Louise shook hands with her and said charmingly, "Thank you, my Sweet." The little mite is a most clever artist, and has drawn quite naturally ever since she could hold a pencil in her baby fingers. The children present, numbering about a score, were given 20 minutes to reproduce from memory a life picture which they had just seen. A small bugler, in his regimentals, had pretended to be seriously wounded. He had sunk into a chair, and then apparently fainted from exhaustion. A dainty little hospital nurse came to the rescue and bent over the wounded warrior. There the two figures stood as if turned to stone, whilst the eyes of the little artists drank in the details of the figure-subject. Suddenly at a given sign the bugler sprang up and scuttled off the platform, followed by the smiling nurse, and the children all started off eagerly to work out their snap-shot sketches, for which three prizes were awarded. The Duchess of Argyll was much amused at the proceedings.

### The Coronation.

Amongst other important details of the Coronation is the manufacture of the carpet to be used on the occasion at Westminster Abbey. The firm at Worcester to whom it has been entrusted were especially selected because they have had long experience in the treatment of such slippery hair as mohair, which has been chosen as the most desirable material for the purpose, owing to the beautiful sheen which it retains even when much trodden upon. Axminster, though beautifully soft, sinks in under pressure, and does not rebound; a carpet composed largely of mohair, though soft, is also of a springy nature, and has not this disadvantage. The colour is deep mandarin blue, with the pattern —consisting largely of roses, shamrock, thistles and lotus flowers—in a paler blue. Wreaths of bay leaves with ribbons, and the badge of the Order of the Garter are also introduced here and there. The total weight will be 2½ tons, the length 725 square yards, and special corners have been woven to avoid "mitres" which would spoil the pattern. After being used to cover the floor of the Abbey on the 26th, the ultimate destination of this wonderful carpet is doubtful. According to an old book kept at the Abbey, which gave directions for the Coronation of Richard II., the portion "under the King's feet as he goeth . . . is always given to the use of the sacrist, and the rest which is outside the church shall be distributed to the poor by the hands of the almoner."

The following Colonial Governors and Premiers will attend the Coronation as royal guests to represent the Colonies:—General Sir Francis Wallace Grenfell representing the Mediterranean, comprising Gibraltar, Malta, and Cyprus; Sir Joseph West Ridgeway, Eastern Colonies and Protectorate, Fiji, and Western Pacific; Sir W. J. Sendall, West Indies, Bermudas, British Honduras, and the Falkland Islands; Sir William MacGregor, West African Colonies and Protectorate; Sir Wilfrid Laurier, Canada; Mr. Edmund Barton, K.C., the Commonwealth of Australia; Mr. R. J. Seddon, New Zealand; Sir John Sprigg, the Cape and St. Helena; Lieut.-Col. Sir Albert H. Hime, Natal; and Sir Robert Bond, Newfoundland. The King has accepted the services of the s.s. *Arabia*, the *Teutonic*, the *Kinfauns Castle*, and the *Akabo*, which have been placed at his disposal by the respective owners for the accommodation and entertainment of His Majesty's personal guests and of the guests of the nation at the Naval Review, to be held at Spithead on Saturday, June 28th.

### Foreign.

The official announcement was made last week that the young Queen of Holland is suffering from typhoid fever, and a notice to this effect is posted at the gate of the palace. This notification is in compliance with Dutch law, which requires that every house in which there is a case of contagious disease shall have an intimation placed outside. Her Majesty is supposed to have contracted the illness as the result of a chill during the recent cold weather. The case is pursuing its usual course, but there is naturally much anxiety in Holland respecting the Queen's condition. Telegrams of inquiry as to the progress of the Royal patient have been received, and continue to be sent, from all foreign Sovereigns and Governments. The Prince Consort celebrates his birthday on Saturday, and many persons called at the palace to enter their names in the visitors' book. But owing to the Queen's illness there was, of course, no special demonstration. Her Majesty's consciousness has been maintained without interruption, and on Tuesday she signed a decree relating to the election of a member of the First Chamber.

### South Africa.

Conferences are still going on in South Africa, but Mr. Balfour has announced that nothing definite in relation to the peace prospects is likely to be known for about three weeks. In the House of Commons on Monday, the Chancellor of the Exchequer, replying to a suggestion of Mr. Gibson Bowles that in the present circumstances he ought to hesitate about increasing taxation, observed, "It is all very well to talk in this way. But I venture to say that nothing can be more premature than the rumours that have appeared in the press on the subject."

### Politics and Society.

As usual, the 19th of April was observed as Primrose Day in memory of the death of Lord Beaconsfield. Both in London and the country the wearing of the flower was general, and was not at all confined to people who sympathised with the politics of the deceased statesman. Thousands visited Parliament Square to see the decoration of the Beaconsfield statue, the most conspicuous feature being a bed of primroses in a shallow box surmounted by a crown, with the inscription "God Defend the Right." This was sent by Mr. E. R. Belilios, a wealthy merchant of Hong Kong, who every year manifests in this way the admiration which he entertained for Lord Beaconsfield.

### The Passing of the Barmaid.

On Tuesday the Glasgow licensing magistrates confirmed their recent action in reference to the employment of barmaids in licensed premises in the city. So far as the public bars of theatres, hotels, and public-houses are concerned, they are to be abolished, but they will still be eligible in connection with bona-fide restaurant business, and the female members of the family of a publican are not included in the proscription. The magistrates agreed to extend the time for dispensing with barmaids until the end of July. Meanwhile, steps are in contemplation to provide other employment for the ex-barmaids.

### For Reading to the Sick.

PEACE BE TO THIS HOUSE.

O CONQUEROR by suffering!
O mighty Victor! glorious King!
From out of pain and sorrow bring
Peace to this house.

"Peace to this house," come, Lord, and say;
Come to us, Lord, and with us stay;
O give, and never take away
Peace from this house.

And when at last our fainting breath
On trembling lips scarce quivereth,
O bring us through the gate of Death,
Lord, to Thine House

To Thine own House in Paradise,
To Thine own House above the skies,
To live the life that never dies,
Lord, in Thine House.

*Bishop Wordsworth.*

We often read of our Saviour visiting the sick. How sweet such moments must have been for those thus privileged.

We may wonder what He would say to a sick person. Sometimes it would be, "I have come to heal thee"; but not always. His visits would not be long, but very sweet and calm, leaving great peace and many a blessing. His manner would be full of sympathy, and His words, we may suppose, would be: "Let not your heart be troubled, nor let it be afraid. You believe in God; believe also in Me. In My Father's house there are many mansions. I go to prepare a place for you, and I will come again, and will take you to Myself, that where I am you also may be; and whither I go you know, and the way you know" (John xiv, 1). And then our Lord would bless the sick person and leave the room. Slowly the power and sweetness of His words come home; they are words that every sufferer should know by heart and make his own by daily meditation. Let us take them one by one.

"Let not your heart be troubled, nor let it be afraid"; a glorious opening, bidding the restless waves of the soul be still under the lash of the storm that is passing over them. A great calm is brought about, and in the clear air all is seen aright, and our Lord adds, "You believe in God, believe also in Me," thus winning the sufferer to Himself by eliciting a humble trust in His sincerity and goodness. "Believe in Me; believe that I do the best for My disciples." "Not for ever, my child, not for long shall this pain continue; you are made for a happy home with Me, and with My own hands, by My own shedding of blood, I have secured and made ready a place for you; the sufferings of this life are not worthy to be compared with the glory to come; be brave and patient on thy bed of sorrow; quickly shall pass the night of suffering here, only keep thy heart ever centred on My Father's house, which lies open to those who are faithful until death."—*Anon.*

Heart-broken and weary, where'er thou mayst be,
There are no words like these words for comforting thee;
When sorrows come round thee like waves of the sea,
The Saviour says graciously, "Come unto Me."

*E. P. Hood.*

### Notes and Queries.

**The Editor is always willing to answer in this column, without any fee, all reasonable questions, as soon as possible.**
**But the following rules must be carefully observed:—**

1. **Every communication must be accompanied by the name and address of the writer.**
2. **The question must always bear upon nursing, directly or indirectly.**

**If an answer is required by letter a fee of half-a-crown must be enclosed with the note containing the inquiry, and we cannot undertake to forward letters addressed to correspondents making inquiries. It is therefore requested that our readers will not enclose either a stamp or a stamped envelope.**

*Address.*

(34) Will you kindly give me the address of a home for nurses from which one can obtain capable hospital-trained women for the sum of one guinea per week?—*A. de B.*

We cannot give private addresses.

*Kidney.*

(35) Will you kindly give me the address of a hospital which treats kidney troubles, and where, for a small fee, advice is given? Is there no place in London where urine can be sent to be tested?—*H. W.*

Any of the larger general hospitals. Any medical man would examine the urine if you wished it, or a complete analysis could be obtained from an analytical chemist.

*Child's Nurse.*

(36) Will you kindly tell me where a nurse, with one year's hospital training, could obtain a few months' training in care of infant from the month, free? She is willing to give her services.—*Nurse W.*

She may hear of what she wants privately or by advertisement, but it is not usual to give such training free.

*Superfluous Hair.*

(37) A patient is extremely anxious to have the hair removed from her upper lip. Will you kindly say what is the best treatment, where she can have it done, and if it is generally successful?—*Nurse F.*

Hair can be removed by electrolysis, but there are other methods. Better consult a skin specialist.

*Private Nursing.*

(38) Will you kindly tell me if there is any way of doing private nursing in India?—*L. R.*

Not unless you go out to take charge of a private patient. You might work into a private connection by first accepting employment under some of the associations.

*Supplementary Training.*

(39) Nurse Patricia has had one year's training in a women's hospital, one year as district nurse, and two years' work in a large convalescent home. She now wishes to join the Naval or Indian Nursing Service but finds that a three years' certificate is necessary. She would be glad to know if it would be possible to get such a certificate as assistant nurse as she does not like the idea of beginning at the beginning again.

As Nurse Patricia has had no training in general nursing she must begin at the beginning again if she wishes to qualify herself for either of the services named.

*Gastritis.*

(40) I wish to consult a physician on gastritis. Can any sister recommend one. I should be very grateful.—*Ignorant Woman.*

We cannot recommend individual practitioners.

*Locality.*

(41) Will you kindly inform me what district within fifty minutes' train journey of London is most suitable for a lady suffering frequently through the winter from neuralgia and muscular rheumatism. She is keenly affected by cold winds and is at present living at Finchley, which does not appear to agree with her at all.—*Cymraes.*

Consult the medical man in charge of the case. Dry, high, sandy or gravelly soils are generally suitable for such cases.

**Standard Books of Reference.**

"The Nursing Profession: How and Where to Train." 2s. net; post free 2s. 4d.
"Burdett's Official Nursing Directory." 3s. net; post free, 3s. 4d.
"Burdett's Hospitals and Charities." 5s.
"The Nurses' Dictionary of Medical Terms." 2s.
"Burdett's Series of Nursing Text-Books." 1s. each.
"A Handbook for Nurses." (Illustrated). 5s.
"Nursing: Its Theory and Practice." New Edition. 3s. 6d.
"Helps in Sickness and to Health." Fifteenth Thousand. 5s.
"The Physiological Feeding of Infants." 1s.
"The Physiological Nursery Chart." 1s.; post free, 1s. 3d.
"Hospital Expenditure: The Commissariat." 2s. 6d.

All these are published by the SCIENTIFIC PRESS, Ltd., and may be obtained through any bookseller or direct from the publishers, 28 and 29 Southampton Street, London, W.C.

### Travel Notes.

**By Our Travelling Correspondent.**

### XCVIII.—HOORN AND HAARLEM.

If you visit Hoorn from Amsterdam the most agreeable way is by steamer, passing Mounikendam and Edam and landing at the beautiful Water Gate, one of the architectural treasures of the world. If you should be staying at Alkmaar or Hoorn itself there is connection between the two places by diligence. Hoorn is on the whole a better resting place than Alkmaar; it was once the capital of North Holland, so that its pretensions to public building are greater, and it has the charm of being actually on the sea. The fleet that sailed with De Ruyter, when he came up the Thames was built here and on the summit of the Water Gate is a vane surmounted by an ancient burnished ship which probably looked down on the departure of that doughty admiral. The unpleasant Van Tromp, too, with his objectionable broom largely recruited his fleet from the hardy fishermen of Hoorn.

Count Horn, whose tragic fate mingled with that of the brilliant Egmont, was born somewhere near to Hoorn, his family name being taken from property adjacent to the city; but all trace of it has disappeared, nor could I gather any reliable information as to its exact position.

### Points of Interest in Hoorn.

First always comes the Water Gate, and try to see this on Thursday, which is market day, because then boats of every sort and kind unload their produce there. Then comes the Stadhuis, with a few really good pictures, the weigh-house early seventeenth century, and the Tribunalshof, the same period. The church is poor, the iconoclasts have been so busy. Schovten is buried in the Groote Kerk, who discovered the passage round Cape Horn and named it after his native city.

One of the most interesting buildings is the St. Jans Gasthuis, an almshouse for old women. Henri Havard, the well-known French writer on Holland, thus describes his visit to it—the victim of a practical joke. "We found our way to the door of this hospital, the victims of the joking and mocking spirit of a grave inhabitant of Hoorn. We were hunting about for artistic bits, when we accosted a good-tempered looking citizen and asked him where anything old and curious was to be seen? 'Antiquities, curiosities? Go to the end of the street and you will find plenty inside that house,' pointing to an ornamented gatehouse. He was laughing at us, it is true, but we saw the beautiful entry to the old women's hospital and did not venture to inquire for the old curiosities within!"

A short distance from Hoorn lies Enkhuizen (rail in about half an hour). There are some picturesque buildings there and it was the birthplace of Paul Potter, but the chief thing to see is the beautiful Roodloft in the Westerkerk, said to surpass all work of its kind in North Holland. From Enkhuizen is the starting point for Friesland, which we shall consider later in the season.

### Haarlem and its Famous Siege.

Haarlem makes an easy excursion from Amsterdam and is full of interest. In the Groote Kerk is the world famous organ, and public recitals are given twice weekly. In the Groote Markt are the Stadhuis and the meat market, the latter a beautiful specimen of Renaissance architecture, but, built in 1602, it cannot have watched the siege which detracts from its interest in my eyes; the church is much older, and could it but speak, might tell us strange things. The weigh-house is worth seeing, and one survivor among the old city gates.

In 1572 began the terrible siege of Haarlem. In Motley's "Rise of the Dutch Republic" there is an account of it so realistic and dramatic, that one almost holds one's breath, as one reads of the dauntless bravery on both sides, the fights in the marshy water and also on the ice, where the Spaniards were naturally at a disadvantage with the skaters. The southerners were much surprised at skates, never having seen, or probably even heard of such things. Alva, however, ordered 7,000 pairs to be made for his soldiers, and they practised daily on the frozen lake of Haarlem till they could meet their foes on more equal terms.

The garrison numbered 4,000 only, but they were of a dogged courage; even the women formed themselves into a regiment under the command of one Kenau Hasselaer. Again and again the besiegers rushed to the assault, but were always repulsed. Boiling oil, boiling water, and red hot coals were thrown on them, as well as hoops steeped in tar and set alight; these were cleverly thrown round their necks by the garrison. The Prince of Orange endeavoured to send in supplies of men and provisions, but never successfully; the first time the force was routed and the officer killed. His bleeding head was thrown over the walls to discourage the besieged. Immediately eleven Spanish prisoners were beheaded, and the barrel containing the heads was thrown into the invading camp with this inscription affixed: "Deliver these heads to Duke Alva, in payment of his tenpenny tax, with one additional head for interest." They were savage times indeed, and in point of humanity there was not much to choose between Dutch and Spaniards. At last, after seven months unsurpassed courage and horrible suffering, Haarlem surrendered; a black flag was flown from the cathedral spire and the starving inhabitants awaited events. A message came from Alva (a liar on every possible occasion) that mercy should be shown and none executed except those whom the citizens deemed worthy of such a fate.

Then began a perfect butchery, beginning with the officers who were all executed together. Eighteen hundred of the original 4,000 garrison remained. Of these 600 were Germans and allowed to march out of the city. The remaining 1,200 were at once massacred, soldiers and civilians, women and children, making altogether about 2,300 victims.

In the wall of the Groote Kerk there is still a cannon ball embedded, which helps one in these peaceful days to realise how different was everything in this now dull old town in the stirring times of 1573. Philip on hearing of the murder of the 2,300 was so much refreshed that he was enabled to rise from a sick bed. "The principal medicine which has cured his majesty," writes a Madrid courtier, "is the joy caused to him by the good news which you have communicated of the surrender of Haarlem!"

### TRAVEL NOTES AND QUERIES.

Dinard or Trouville (Centaur).—They are both very gay, and in that respect there is not much to choose between them; but Dinard has much more interesting surroundings, which are far less conventional than about Trouville. Brittany still retains some of its primitiveness, and is more interesting than the more cultivated and commercial Normandy. For expense the two places are about the same, perhaps Trouville may be a trifle the dearer of the two. At Dinard you are near Dinan, St. Servan, St. Malo, St. Briac, and not very far from Roscoff, etc. At Trouville you have only Honfleur and Deauville.

Rouen for a Fortnight (Flauto Magico).—I think you could not do better than go to the Hôtel de la Poste close to the Post Office. If you were tired of the town, there are endless excursions to be made, both long and short, especially on the Seine. How would it be, if your companion does not like town life, to spend only one week in Rouen, which would be ample time to study the churches, and the other at Caudebee on the Seine, which is a charming place and a very good centre from which to explore the river, Jumièges, etc.?

## Figures and Tables

**Figure f1:**
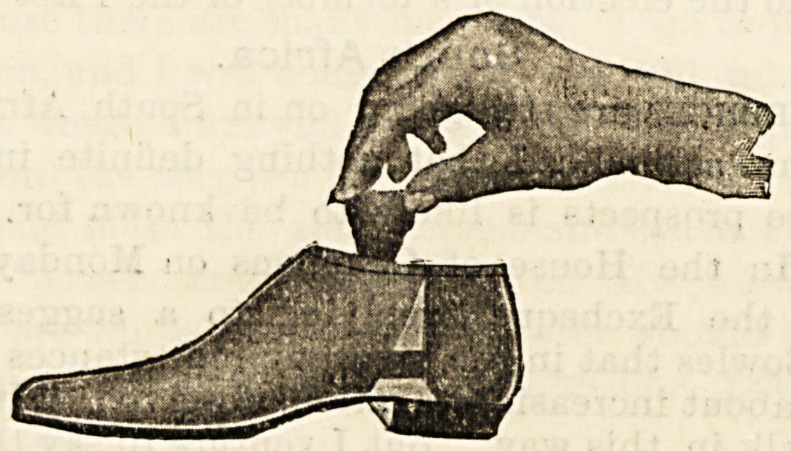


**Figure f2:**